# Wideband miniaturized patch radiator for Sub-6 GHz 5G devices

**DOI:** 10.1016/j.heliyon.2021.e07931

**Published:** 2021-09-03

**Authors:** Ankush Kapoor, Ranjan Mishra, Pradeep Kumar

**Affiliations:** a Department of Electronics and Communication Engineering, Jawaharlal Nehru Government Engineering College, Sundernagar, Mandi, India; b Department of Electrical and Electronics Engineering, University of Petroleum and Energy Studies, Dehradun, India; c Discipiline of Electrical, Electronic and Computer Engineering, University of KwaZulu-Natal, Durban, 4041, South Africa

**Keywords:** Miniaturization, Elliptical slotted semi-circular patch radiator (ESSPR), Sub-6 GHz bands, Semi-circular patch, Slotted antenna

## Abstract

In this article, the compact wideband elliptically slotted semi-circular patch radiator with the defected ground structure for sub-6 GHz applications is designed and developed. The proposed miniaturized patch radiator offers flexibility in adjusting the band of operation by varying the slot dimensions. The effective size reduction is achieved by comparing different iterations in the process of designing and the size of the regular circular-shaped radiator is reduced into the semi-circular radiating patch. The impact of the variation of effective radius of the semi-circular patch, major-axis radius of the elliptical slot, ground plane length, and feed line width is investigated. The size of the proposed radiator is 23.885 × 23.885 × 1.405 mm^3^. This compact structure manifests the wide bandwidth of 2140 MHz (3.2 GHz–5.34 GHz) with 50% of fractional bandwidth (FBW). The measured results show good agreement with the simulated results. The various parameters validate the utility of the radiator in the C band of super-high frequency (SHF) spectrum and n77 (3.3 GHz–4.2 GHz), n78 (3.3 GHz–3.8 GHz), and n79 (4.4 GHz–5 GHz) bands of the frequency range 1 (FR1) of the sub-6 GHz 5G spectrum.

## 1 Introduction

Printed patch radiators were originally presented in the 1950s, but they did not achieve success until the 1970's when they were propelled by the rapid advancement of the integration technology with digitization of wireless information to be transferred. However, even though these antennas are compact, easy to fabricate with low production cost, and are easy to integrate with radio frequency circuits. These antennas also suffer with low fractional bandwidth (FBW = 7%) which proves their incompatibility with the current wireless technologies. To address this limitation, substantial research has been conducted in the last two decades and new novel geometries have been discovered to enhance the bandwidth. This necessitated the discovery of the physical mechanisms regulating the radiative processes occurring in the antennas. 5G technology has opened a way of different research perspectives on its horizon. By the year 2025, the expected number of operational smart devices will be approximately 6.5 billion, which is quite high as compared to the bank accounts (5.5 billion) or many operational landlines (3.0 billion) [1]. To fulfill the demands of the emerging wireless devices and for achieving an ultra-fast transmission rate (peak data rate ≥20 Gbps), low latency (≤1 ms), densely populated connections, and higher mobility (≥500 km/h), the 5G wireless communication systems were deployed in the first quarter of 2020 [1]. The estimation of the 5G device connections is expected to reach 577 million by 2023 as compared to just 5 million that was reported in 2019 [2]. The frequency bands for 5G deployment were allocated comprising of three bands which are low-band (up to 1GHz), mid-band (sub-6 GHz), and high-band (mm-Wave) [3, 4, 5]. The focus of the researchers is for the design of miniaturized patch radiators, which can be a feasible choice for integration with the existing 4G technologies and budding sub-6 GHz 5G cellular networks. Numerous studies have been undertaken in recent years for antenna miniaturization. Slot antennas have been used in various applications as they offer a range of advantages. Various slot shapes engraved on the antenna are studied in which they provide an added advantage of providing desired bandwidth adjustments [6, 7, 8, 9, 10]. An ultra-wideband antenna based on a modified ground plane is presented for being operated in the sub-6 GHz 5G bandwidth of 2.32 GHz–5.24 GHz [11]. Also, many other shapes of the slots embedded in the antenna geometry targeting multiband operation [12], T-shape slots [13], L-shaped slots [14], elliptical/circular slots [15], semi-circular slots [16], C-shaped slots [17], hexagonal slots [18, 19] and so forth have been proposed. The modified ground plane of microstrip antennas helps in achieving dual-band [20], wide-bandwidth [21], etc. In [22, 23], a multiband circularly polarized slot antenna is designed and fabricated. The incorporation of the modified ground plane structure helps to achieve circularly polarized characteristics and it also improves the axial ratio bandwidth by using a D-shaped radiator. In adding to these advantages a new slot antenna design for dual-band operation is proposed in which two adjunct arc-shaped slots are added to two main semi-circular slots etched on the ground plane to achieve the desired frequency bands [24]. The study of a slotted semi-circular antenna is taken further by engraving a floral shape slot and analyzing it in a detailed manner [25]. The key design advantages of adding these slots are reduced size, simple configuration, adequate gain etc. A wideband antenna for sub-6 GHz applications is proposed in [26], but it is of large size. In [27], the researchers have reported the benefit of engraving an elliptical slot but still its large size offers limited usage. In [28], the circular patch with the defected ground plane is developed for ultra-wideband applications but offers low average gain for the sub-6 GHz 5G applications.

Inspired from the above reported research articles, this article presents a novel compact patch radiator achieving wide bandwidth in sub-6 5G wireless applications. A semi-circular patch having elliptical slot, fed at an apex by microstrip feed line, exhibiting radiation in n77 (3.3 GHz −4.2 GHz), n78 (3.3 GHz −3.8 GHz) and n79 (4.4 GHz–5 GHz) bands of frequency range 1 (FR1) in sub- 6 GHz spectrum is proposed. The effect of inserting the slot and reducing the size of the ground plane is observed, which indicates that it helps in achieving the wide bandwidth. The design iterations consider the circular patch of radius (r), which is evaluated and then modified into a semicircular shape. The effect of inserting different shapes of slot structures is investigated and output performance characteristics of wideband operation with compact size enabled us to insert the elliptical slot in the designed semicircular patch. Thus, an elliptical slotted semicircular patch radiator (ESSPR) is designed and developed for sub-6 GHz 5G communications. Rest of the paper is organized as follows. Section 2 presents the design and geometrical configuration of the proposed ESSPR. The simulated results of the ESSPR are presented in section 3. The fabrication, measured results, validation of measured results with simulated results, and comparison of proposed ESSPR with existing designs are discussed in section 4, whereas section 5 presents the conclusion of the work.

## 2 Design and geometry of the proposed radiator

**Figure 1:**
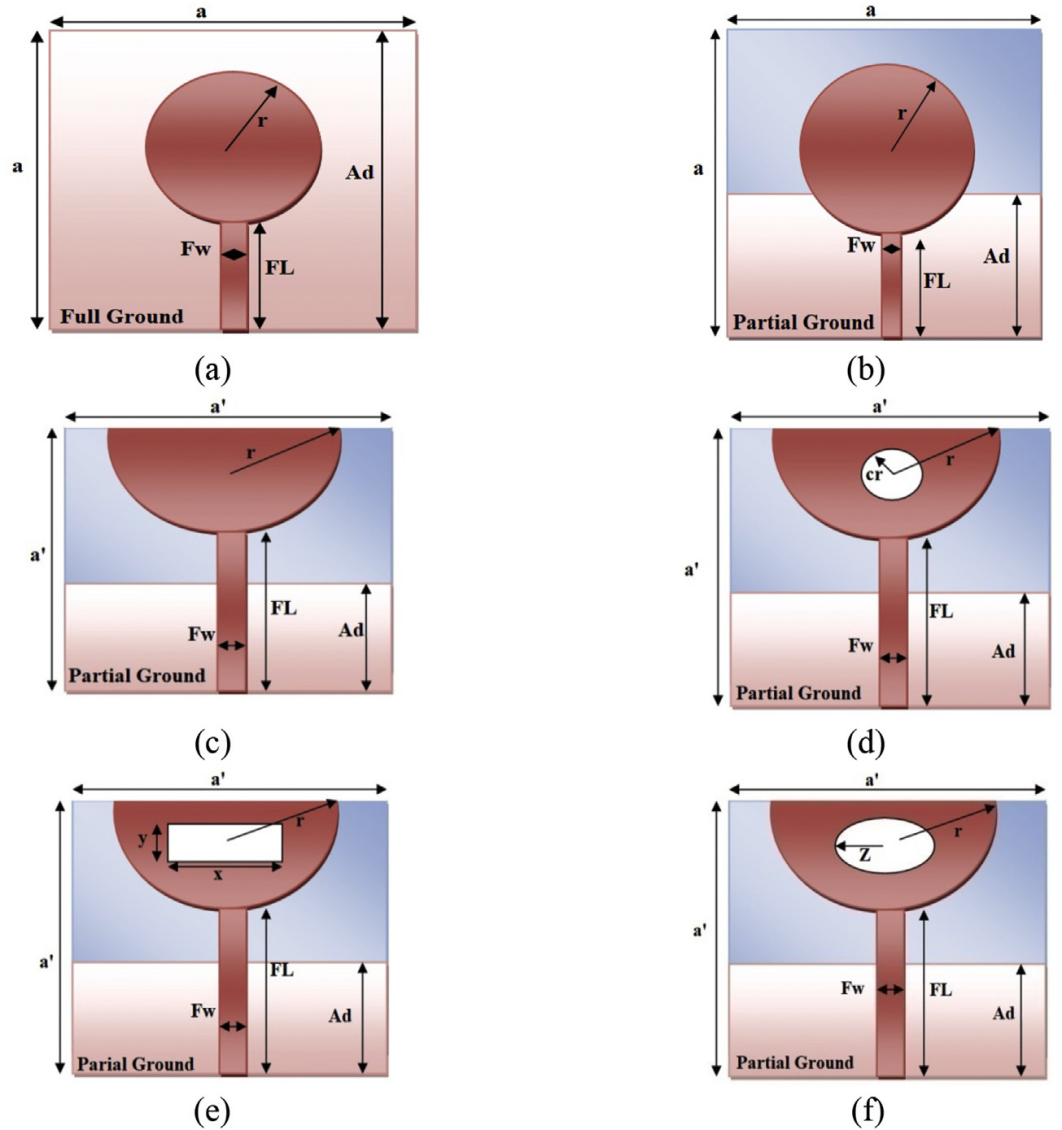
Evolution stages of the proposed ESSPR geometry, (a) Radiator1, (b) Radiator2, (c) Radiator3, (d) Radiator4, (e) Radiator5, and (f) Radiator6 (ESSPR).

It is simpler to design the circular and semi-circular microstrip patch radiators as compared to the other geometries as only one design parameter i.e. patch radius (r). Since, the circular geometry provides the minimal footprint area, hence a circular patch radiator is initially constructed and is taken as a reference design. The different iterations of the antenna are targeted to be operated at the center frequency (f_c_) of 4.27 GHz in the sub- 6 GHz frequency range from 3.20 GHz to 5.34 GHz. The Flame retardant 4 (FR4) substrate is chosen with the relative permittivity (ε_r_) of 4.4 and an overall thickness (t) of 0.02λ. The design steps which are opted to develop the ESSPR are given in Figure 1 and are elaborated as follows:

### 2.1 Step-1 (Radiator1)

Initially, a reference antenna or Radiator1 consisting of a microstrip line fed circular patch radiator with full ground plane is designed. The actual patch radius (r) is evaluated by [23]:

(1) r=F{1+2tπεrF[ln(πF2t)+1.7726]}12

In the above Eq. (1), the constant F can be evaluated by using Eq. (2):

(2) $$\text{F}=\frac{8.791\times 10^{9}}{{\text{f}}_{\text{c}}\sqrt{{\text{ε}}_{\text{r}}}}$$

In the above formulae, the unit of operating center frequency (f_c_) is in Hertz (Hz) and the unit of the overall thickness of the substrate (t) is in centimeters (cm). The values substituted in Eq. (1) for the parameters are as f_c_ = 4.27 × 10^9^ Hz, ε_r_ = 4.4 and t = 0.02λ. Using these, the value of F comes out to be 1.0135 and r to be 0.97 cm i.e. 0.13λ. The dimension of the ground is taken as 0.42λ, where λ is the wavelength corresponding to center frequency.

### 2.2 Step-2 (Radiator2)

In this step, the concept of a defected ground substrate (DGS) is being used and the dimension of the ground plane is optimized to a value at which we get considerable performance in the desired sub-6 GHz frequency range. The concept of DGS is already proven by many researchers to provide wider bandwidth and many DGS shapes have already been suggested in the literature. The advantage of reducing the dimension of the ground plane is that it reduces inductive coupling of the ground plane with the radiator and hence it helps to increase the bandwidth as the energy stored in the substrates gets reduced and thus it decreases the quality factor (Q factor) and increases the bandwidth [29].

### 2.3 Step-3 (Radiator3)

In the next step, the circular patch is converted into the semi-circular patch radiator. The radius (r) as calculated from Eqs. (1) and (2) is maintained at the same value and the effective dimensions of the substrate are optimized and reduced to attain miniaturization while maintaining and even improving the output characteristics of the designed structure. The length of the substrate is 0.34λ. The dimension of the ground plane is optimized as 0.17λ. The dimensions of the Radiator3, shown in Table 1, are used for attaining the desirable characteristics as given in Table 2. The antenna radiates with an adequate bandwidth. The value of gain and directivity motivates us to modify this design for getting the better performance.

**Table 1:** Dimensions of the various configurations.

Parameters	Dimensions (λ is the wavelength at the center frequency)					
	Radiator1	Radiator2	Radiator3	Radiator4	Radiator5	Radiator6
Overall thickness (t)	0.02λ	0.02λ	0.02λ	0.02λ	0.02λ	0.02λ
Substrate side length	a = 0.42λ	a = 0.42λ	a’ = 0.34λ	a’ = 0.34λ	a’ = 0.34λ	a’ = 0.34λ
Dimensions (W × L × h)	0.42λ × 0.42λ × 0.02λ	0.42λ × 0.42λ × 0.02λ	0.34λ × 0.34λ × 0.02λ	0.34λ× 0.34λ ×0.02λ	0.34λ × 0.34λ × 0.02λ	0.34λ × 0.34λ × 0.02λ
Circular patch radius (r)	0.13λ	0.13λ	0.13λ	0.13λ	0.13λ	0.13λ
Circular Slot Radius (cr)	-	-	-	0.04λ	-	-
Rectangular slot length (x)	-	-	-	-	0.14λ	-
Rectangular slot width (y)	-	-	-	-	0.05λ	-
Major radius of slot (Z)	-	-	-	**-**	**-**	0.04λ
Semi-minor axis of slot	-	-	-	**-**	**-**	0.02λ
Feed line length (FL)	0.22λ	0.22λ	0.22λ	0.22λ	0.22λ	0.22λ
Feed line width (F_W_)	0.04λ	0.04λ	0.04λ	0.04λ	0.04λ	0.04λ
Ground length (Ad)	0.42λ	0.21λ	0.17λ	0.17λ	0.17λ	0.17λ

**Table 2:** Antenna parameters of various configurations.

Output characteristics	Value					
	Radiator1	Radiator2	Radiator3	Radiator4	Radiator5	Radiator6
Highest frequency (f_H_)	NR	NR	5.67 GHz	4.78 GHz	4.47 GHz	5.34 GHz
Lowest frequency (f_L_)	NR	NR	3.26 GHz	3.62 GHz	3.56 GHz	3.20 GHz
Bandwidth (f_H_ – f_L_)	NR	NR	2410 MHz	1160 MHz	910 MHz	2140 MHz
Gain (dB)	-	-	1.81	2.98	2.78	2.76
Directivity (dB)	-	-	2.11	3.17	2.95	2.98

NR- Not Radiating.

### 2.4 Step-4 (Radiator4)

The design geometry is modified by inserting slots in the patch radiator for achieving miniaturization with improved performance characteristics. The slot in the patch helps to get the smaller microstrip antenna with improved bandwidth and efficiency. A circular slot with the slot radius of 0.04λ is inserted at the center of the patch radiator. The effect of the slot is visible with the data reported in Table 2. Here, the values of gain and directivity are enhanced along with the adequate bandwidth. However, the bandwidth of Radiator4 is not as good as retrieved in Step-3.

### 2.5 Step-5 (Radiator5)

The optimized design, which is achieved by the Radiator3, is utilized and a rectangular slot is inserted into the semicircular patch for achieving the miniaturization. The dimensions of the inserted rectangular slot are kept such that the length of the slot is 0.14λ and the width of the slot as 0.05λ. The radius (r) of the patch radiator, calculated using Eqs. (1) and (2), is used in the design of Radiator3. The performance characteristics are reported in Table 2, which indicate that although the gain and directivity are better than Radiator3, however, Radiator4 gives better gain and directivity as compared to Radiator3.

### 2.6 Step-6 (Radiator6)

In this step, the elliptical slot is inserted into the semicircular patch. The advantage of elliptical slot for achieving the miniaturization is that it helps in reducing the radiating area with adequate characteristics and is also insensitive to the polarization. The dimensions of the elliptical slot are directly related to the lowest transmission zero frequency of the impedance bandwidth. An empirical formula to extract the lowest transmission zero frequency of the elliptical disc monopoles is given by [30]:

(3) $${\text{f}}_{\text{l}}=\frac{30\times 0.24}{\text{H}+\text{r}}$$

where f_l_ (in GHz) denotes the lower transmission zero frequency, H is the effective height of the elliptical disc to be engraved and r denotes the equivalent radius of the elliptical cylinder. Further in [31], Eq. (3) has been modified and is formulated as:

(4) $${\text{f}}_{\text{l}}=\frac{30\times \text{X}}{\text{H}+\text{r}}$$

In equation (4), H and r are in centimeters and f_l_ is in GHz. The term X denotes the element factor which is equal to 0.32 for the elliptical slot and 0.35 for the circular slot. The semi-major axis and semi-minor axis of the elliptical slot are 0.04λ and 0.02λ, respectively. The performance characteristics are reported in Table 2. From Table 2, it is noticeable that an elliptical slot-based semicircular patch radiator proves to be the best candidate for achieving wideband radiation characteristics with reasonable gain and directivity. Thus, the elliptical slotted semicircular patch radiator (ESSPR) is fabricated and its detailed analysis is presented.

The procedure followed to develop the geometry of the proposed elliptical slot semicircular patch radiator is depicted in Figure 1. A SMA connector is connected to the transmission line and the ground plane. Table 1 gives a detailed explanation of the proposed antenna's design specifications. A semicircular disc patch is analysed by treating it as equivalent to the rectangular patch radiator with the dimensions ‘${\text{L}}_{\text{e}}\times {\text{W}}_{\text{e}}$’ [32], where ${\text{L}}_{\text{e}}=2\text{a ​}$ and ${\text{W}}_{\text{e}}=\text{a}/2$. The resonance frequency of a semicircular disc patch antenna may be computed using Eq. (5) [33, 34]:

(5) $${\text{f}}_{\text{r}}=\frac{{\text{k}}_{\text{pq}}\text{C}}{2\text{π}{\text{a}}_{\text{e}}\sqrt{{\text{ε}}_{\text{e}}}}$$

where ${\text{ ​k}}_{\text{pq}}$ is the p^th^ zero root of the derivative of bessel function of the order q, c is the velocity of light, ε_e_ is the effective dielectric constant of the substrate and the effective radius of the semicircular disk patch is given by Eq. (6) [34]:

(6) $${\text{a}}_{\text{e}}=\sqrt{\frac{{\text{L}}_{\text{e}}{\text{W}}_{\text{e}}}{\text{π}}}$$

**Figure 2:**
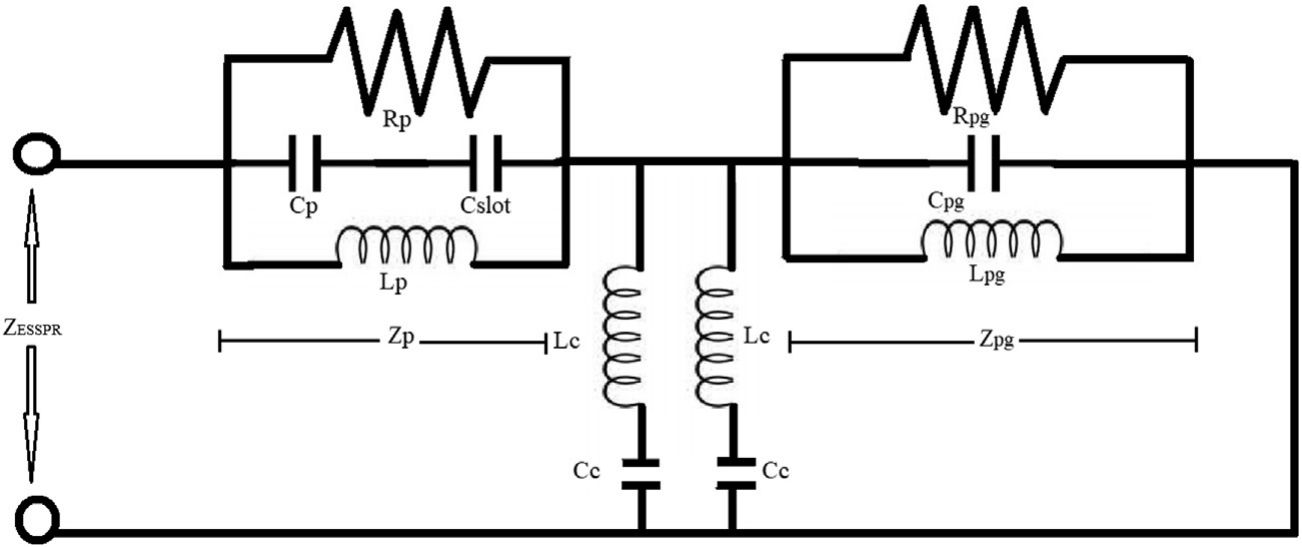
Equivalent circuit of the proposed ESSPR.

The effective radius (${\text{a}}_{\text{e}}$) of the semicircular disk patch padiator is derived by equating the area of the semicircular patch to the equivalent rectangular patch of dimension (${\text{L}}_{\text{e}}\times {\text{W}}_{\text{e}}$), where ${\text{L}}_{\text{e}}$and ${\text{W}}_{\text{e}}$ are the rectangular patch's effective length and width, respectively [33]. The equivalent circuit of the final proposed elliptical slotted semicircular patch radiator is shown in Figure 2 with circuit parameters i.e. patch resistance (${\text{R}}_{\text{p}}$), patch inductance (${\text{L}}_{\text{p}}$), patch capacitance (${\text{C}}_{\text{p}}$), slot capacitance (${\text{C}}_{\text{slot}})$, partial ground resistance (R_pg_), partial ground inductance (L_pg_), partial ground capacitance (C_pg_) and lumped elements due to coupling. These parameters are calculated by using mathematical formulations as given in Eqs. (7), (8), and (9) below [34, 35]:

(7) $${\text{C}}_{\text{P}}=\frac{{\text{ε}}_{\text{o}}{\text{ε}}_{\text{eff}}{\text{L}}_{\text{e}}{\text{W}}_{\text{e}}}{\text{h}}$$

(8) $${\text{L}}_{\text{P}}=\frac{1}{{\text{ω}}_{\text{p}}^{2}{\text{C}}_{\text{p}}}$$

(9) $${\text{R}}_{\text{P}}=\frac{{\text{Q}}_{\text{r}}}{{\text{ω}}_{\text{p}}{\text{C}}_{\text{p}}}$$

where L_e,_ W_e_, ***ε***_ref_ and ω_p_ denote the length of the rectangular patch, the width of the rectangular patch, the effective permittivity of the medium and the angular frequency, respectively.

The value of ${\text{Q}}_{\text{r}}$can be evaluated using Eq. (10) [34, 35]:

(10) $${\text{Q}}_{\text{r}}=\frac{\text{c}\sqrt{{\text{ε}}_{\text{eff}}}}{{\text{f}}_{\text{p}}\text{h}}$$

where c, h and ${\text{f}}_{\text{p}}$ denote the velocity of light in free space, the height of the substrate and the resonant frequency of operation, respectively. The impedance of the patch is given in Eq. (11):

(11) $${\text{Z}}_{\text{p}}=\frac{1}{\frac{1}{{\text{R}}_{\text{p}}}+\frac{1}{{\text{jwL}}_{\text{p}}}+\text{jω}{\text{C}}_{\text{p}}}$$

The equivalent impedance (${\text{Z}}_{\text{pg}}$) of partial ground plane is considered to be a parallel combination of the inductance (${\text{L}}_{\text{pg}}$), capacitance (${\text{C}}_{\text{pg}})$, and resistance (${\text{R}}_{\text{pg}}$) and is computed by using Eq. (12) [36]:

(12) $${\text{Z}}_{\text{pg}}=\frac{1}{\frac{1}{{\text{R}}_{\text{pg}}}+\frac{1}{{\text{jwL}}_{\text{pg}}}+\text{j}{\text{ω}}_{\text{pg}}}$$

It's worth noting that the two resonant circuits, one of which is the lumped equivalent circuit of the rectangular patch radiator and the other is the partial ground plane as illustrated in Figure 2, are linked together by a mutual inductance (${\text{L}}_{\text{C}}$) and mutual capacitance (${\text{C}}_{\text{C}}$). The capacitance due to engraving of a slot is added in series with the overall capacitance of the patch. In the proposed design, an elliptical slot is added to the semicircular patch radiator. The different iterations, comprising of a circular, rectangular and an elliptical slot, are given in Figure 1. The output characteristics retrieved from the different iterations are mentioned in Table 2, which clearly indicates that an elliptical slot is best to achieve wide bandwidth among all slotted designs as illustrated with adequate values of gain and the directivity.

The resonant frequency (f_R_) of any tuned circuit is given in Eq. (13) [32, 33, 34]:

(13) $${\text{f}}_{\text{R}}=\frac{1}{2\text{π}\sqrt{{\text{L}}^{\prime }{\text{C}}^{\prime }}}$$

where ${\text{L}}^{\prime }={\text{ ​L}}_{\text{P}}$ and ${\text{C}}^{\prime }=\frac{1}{\frac{1}{{\text{C}}_{\text{P}}}+\frac{1}{{\text{C}}_{\text{slot}}}}$.

It may be noted that by engraving a slot within the patch antenna radiator, the value of overall capacitance reduces, which in turn helps to increase the resonant frequency of operation as per Eq. (13). It may be noted that the capacitance offered by a rectangular slot is minimum and that to offered by an elliptical slot is maximum. Hence, the overall capacitance of the final design consisting of an elliptical slot is least among the other circular and rectangular slotted designs. It results in the largest bandwidth offered by an elliptical slotted semicircular patch antenna radiator as compared with other slotted structures. The effect in the output characteristics is visualized in Table 2 which validate the theoretical interpretations. The equivalent impedance as a result of coupling between the ground plane and the patch antenna radiator is determined by using Eq. (14) [36]:

(14) $${\text{Z}}_{\text{c}}=\left(\text{jω}{\text{L}}_{\text{c}}+\frac{1}{\text{jω}{\text{C}}_{\text{c}}}\right)$$

where C_c_ and L_c_ depict the mutual capacitance and the inductance of the two resonant circuits, respectively, as illustrated in Figure 2 and are given in Eqs. (15) and (16) [34]:

(15) $${\text{L}}_{\text{c}}=\frac{\text{X}\left(\text{L′}+{\text{L}}_{\text{pg}}\right)+\sqrt{{\text{X}}^{2}{\left(\text{L′}+{\text{L}}_{\text{pg}}\right)}^{2}+4\text{C}{\text{′}}^{2}\left(1-{\text{X}}^{2}\right)\text{L′}{\text{L}}_{\text{pg}}}}{2\left(1-{\text{X}}^{2}\right)}$$

(16) $${\text{C}}_{\text{c}}=\frac{-\left(\text{C′}+\text{X}\right)+\sqrt{{\left(\text{C′}+\text{X}\right)}^{2}-4\text{C′X}\left(1-{\text{X}}^{-2}\right)}}{2}$$

where $\text{X}=\frac{1}{\sqrt{{\text{Q}}_{1}{\text{Q}}_{2}}}$ , and Q_1_, Q_2_ are the quality factors of the both resonant circuits. The impedance of the proposed ESSPR is given in Eq. (17):

(17) $${\text{Z}}_{\text{ESSPR}}={\text{Z}}_{\text{pg}}+\frac{{\text{Z}}_{\text{p}}{\text{Z}}_{\text{c}}}{{\text{Z}}_{\text{p}}+{\text{Z}}_{\text{c}}}$$

**Figure 3:**
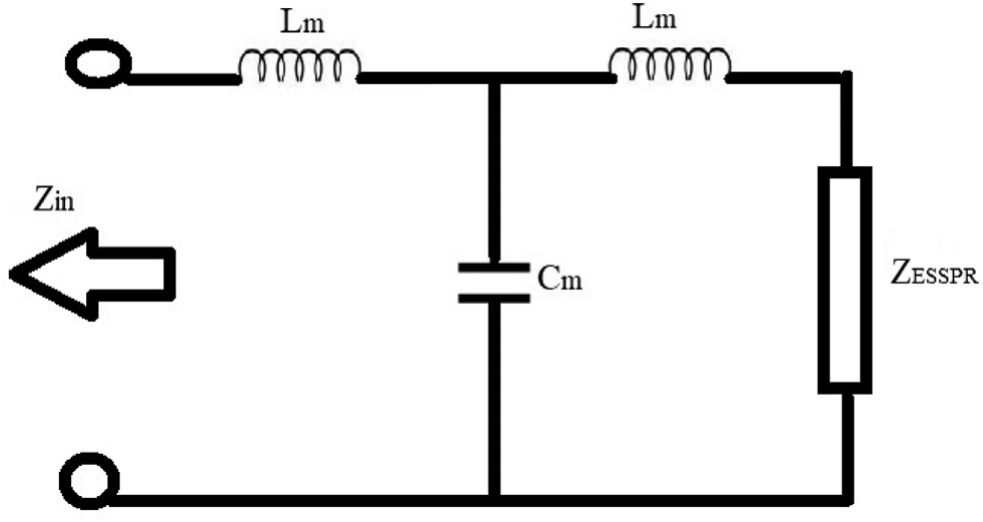
Equivalent circuit of the proposed ESSPR with microstrip transmission line feed.

The designed patch radiator consists of a simple microstrip line feed which is considered as parallel combination of impedance Z_ESSPR_, inductance (L_m_) and the capacitance (C_m_) as shown in Figure 3. The term Z_ESSPR_ is considered to be the impedance of a rectangular patch radiator including partial ground plane, and the values of L_m_ and C_m_ are calculated by using Eqs. (18) and (19) [34]:

(18) $${\text{C}}_{\text{m}}={\text{W}}_{1}\left[\left(10.1\text{ ​log ​}{\text{ε}}_{\text{reff}}+2.33\right)\times \frac{{\text{W}}_{1}}{{\text{W}}_{2}}-12.6\text{ ​log ​}{\text{ε}}_{\text{reff}}-317\right]\text{pF}$$

(19) $${\text{L}}_{\text{m}}=\text{h}\left[40.5\left(\frac{{\text{W}}_{1}}{{\text{W}}_{2}}-1.0\right)-75\text{ ​log}\left(\frac{{\text{W}}_{1}}{{\text{W}}_{2}}\right)+0.2{\left(\frac{{\text{W}}_{1}}{{\text{W}}_{2}}-1.0\right)}^{2}\right]\text{nF}$$

where W_1_ and W_2_ denote the width of the patch and the width of the microstrip feedline, respectively.

Also, the total input impedance is extracted by using Eq. (20) for the proposed designed antenna as [35]:

(20) $${\text{Z}}_{\text{in}}={\text{Z}}_{\text{Lm}}+\frac{\left(\frac{1}{\text{Zcm}}\right)\left({\text{Z}}_{\text{Lm}}+{\text{Z}}_{\text{ESSPR}}\right)}{\left(\frac{1}{\text{Zcm}}\right)+\left({\text{Z}}_{\text{Lm}}+{\text{Z}}_{\text{ESSPR}}\right)}$$

where Z_Lm_ = jωL_m_ and Z_cm_ = jωC_m_.

Hence, various characteristics such as reflection coefficient, return loss and voltage standing wave ratio are evaluated by using Eqs. (21), (22), and (23):

Reflection coefficient,

(21) $$\text{Γ}=\frac{\text{Z}-{\text{Z}}_{\text{in}}}{\text{Z}+{\text{Z}}_{\text{in}}}$$

where Z denotes the overall impedance of the microstrip line feed and is taken as 50 Ω. The relation between the return loss (RL) and reflection coefficient is given by:

(22) RL=−20log|Γ|

Voltage standing wave ratio (VSWR) can be calculated using:

(23) $$\text{VSWR}=\frac{1+\left|\text{Γ}\right|}{1-\left|\text{Γ}\right|}$$

**Figure 4:**
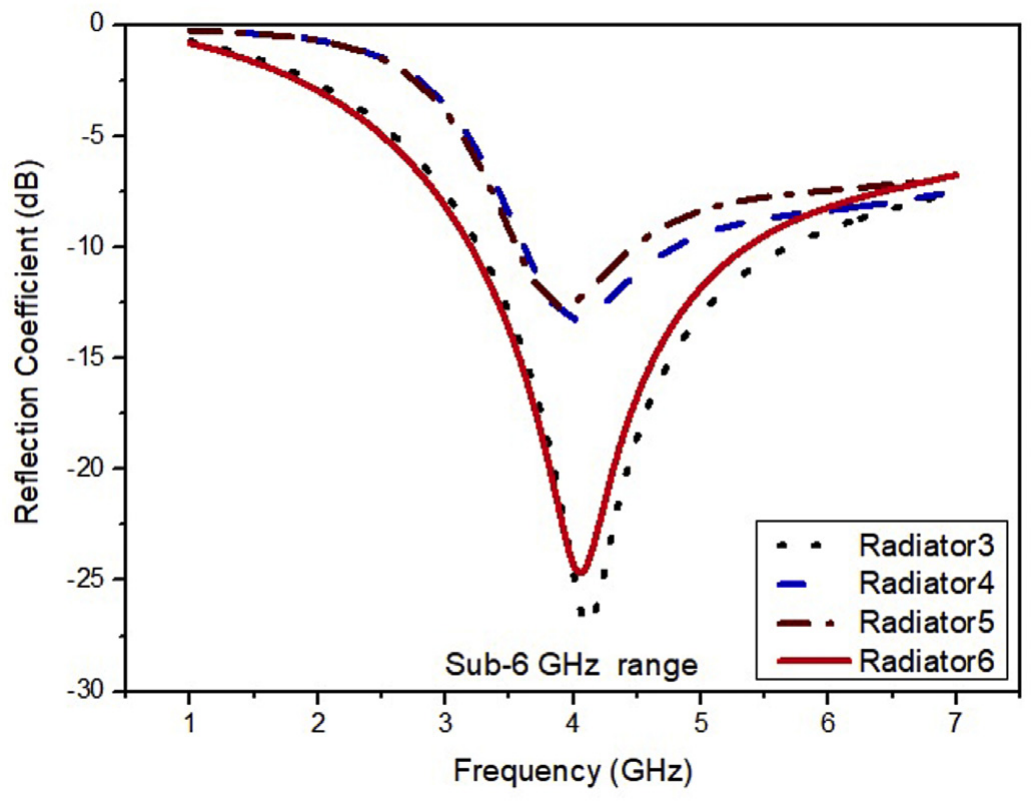
Reflection coefficient of the Radiator3, Radiator4, Radiator5 and Radiator6.

**Figure 5:**
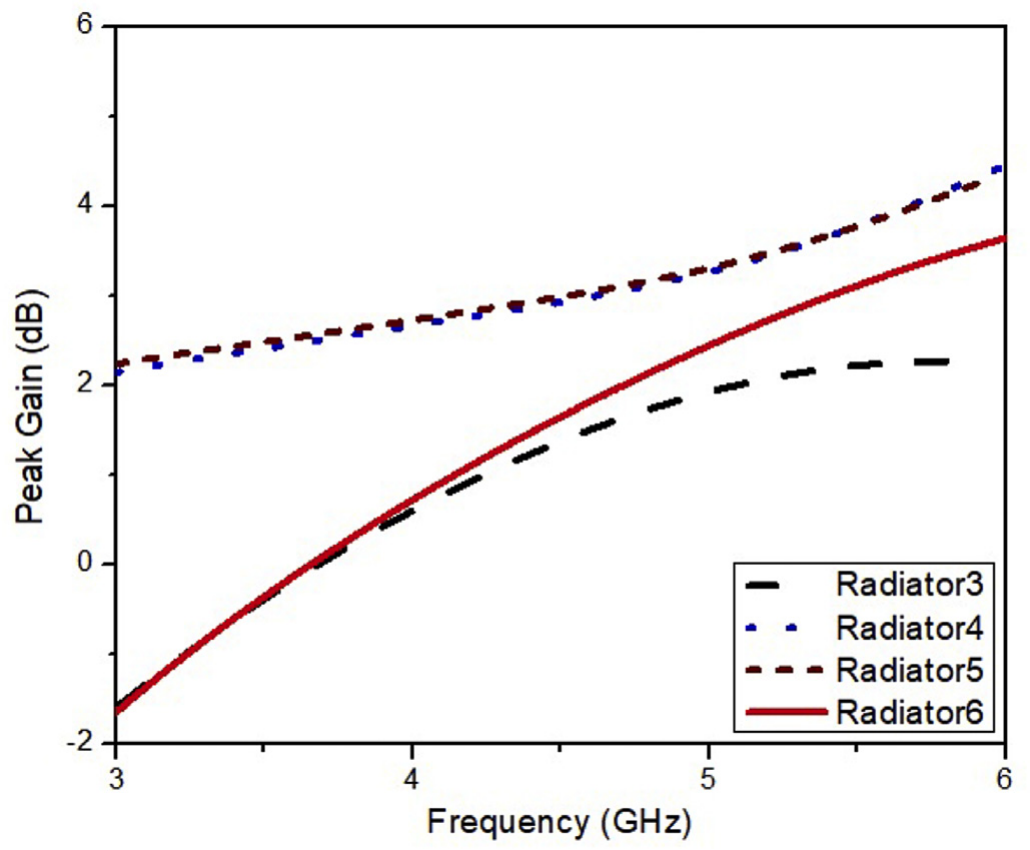
Gain versus frequency plot for the Radiator3, Radiator4, Radiator5 and Radiator6.

**Figure 6:**
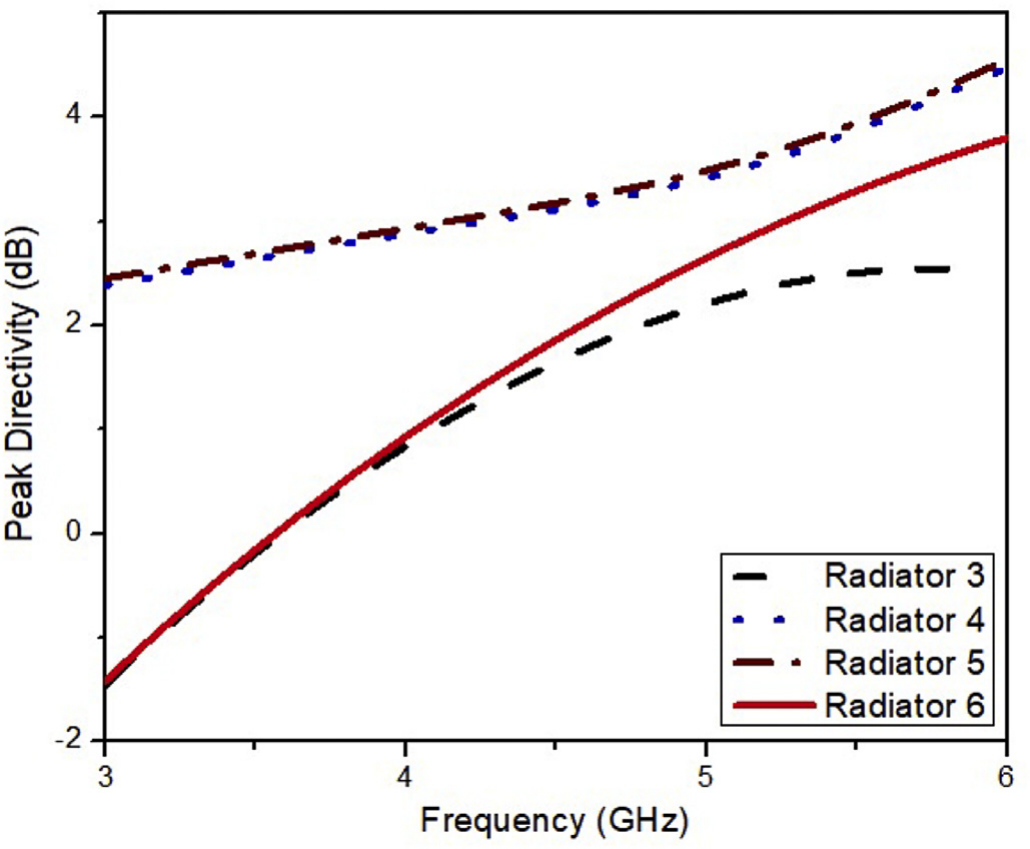
Directivity versus frequency plot for the Radiator3, Radiator4, Radiator5 and Radiator6.

It can be noted from Figure 4 that the bandwidth of the ESSPR covers the n77, n78, and n79 bands. The Radiator6 has explicit features of simple design, miniaturized structure (0.34λ× 0.34λ×0.02λ), and the simpler fabrication process. The comparative analysis of gain and directivity is illustrated in Figure 5 and Figure 6, respectively, which indicates that the proposed ESSPR is the best candidate to be used in sub-6 GHz devices due to its wide bandwidth and compact size.

## 3 Simulated results of the ESSPR

**Figure 7:**
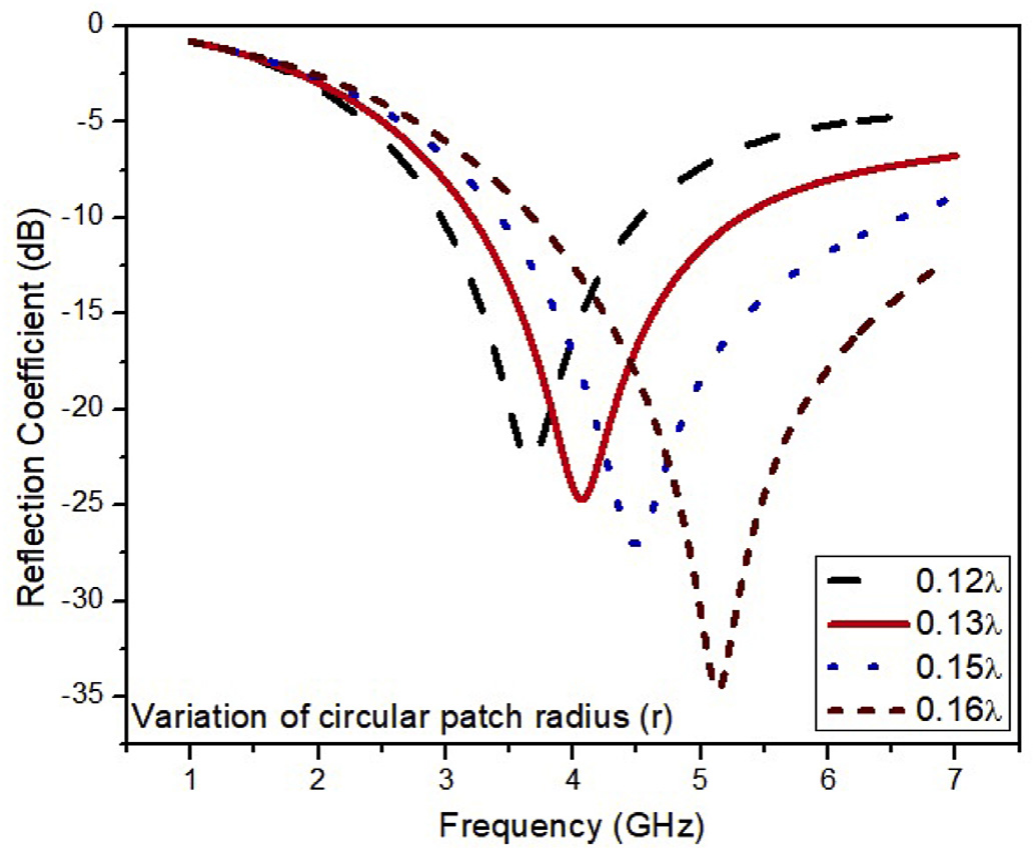
Effect of radius (r) of semi-circular patch on the reflection coefficient of the ESSPR.

**Figure 8:**
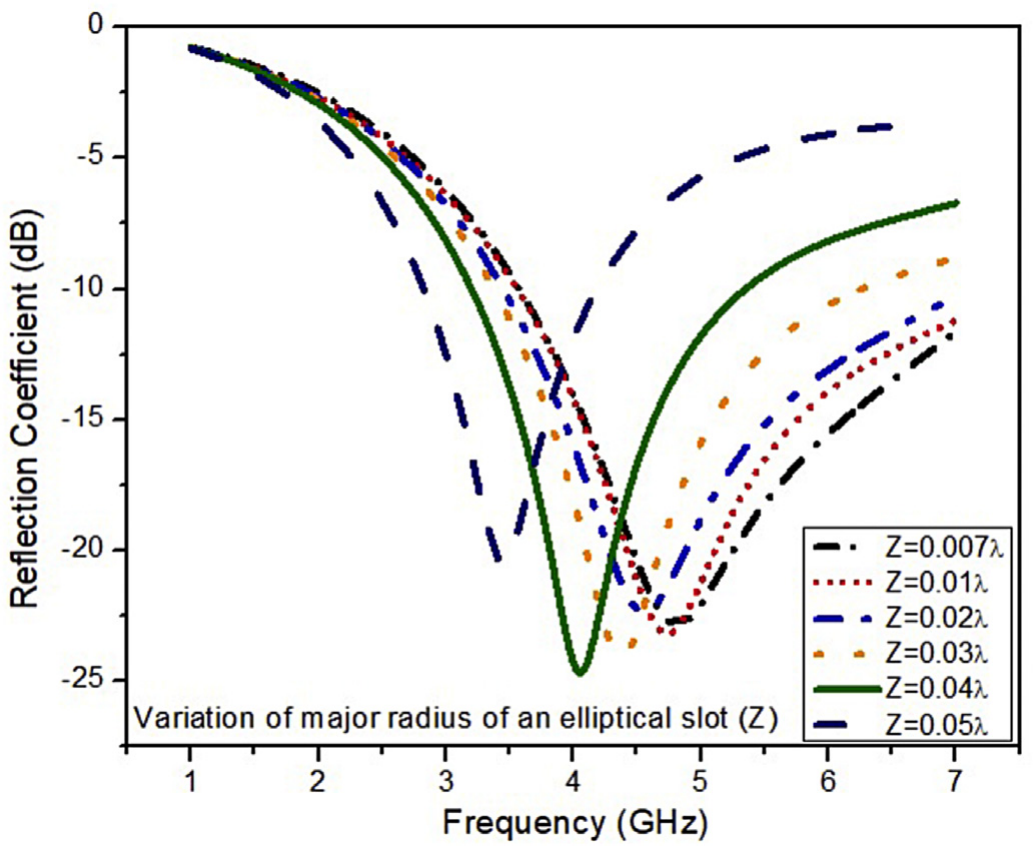
Effect of the major-axis radius (Z) of the elliptical slot on reflection coefficient of the ESSPR.

**Figure 9:**
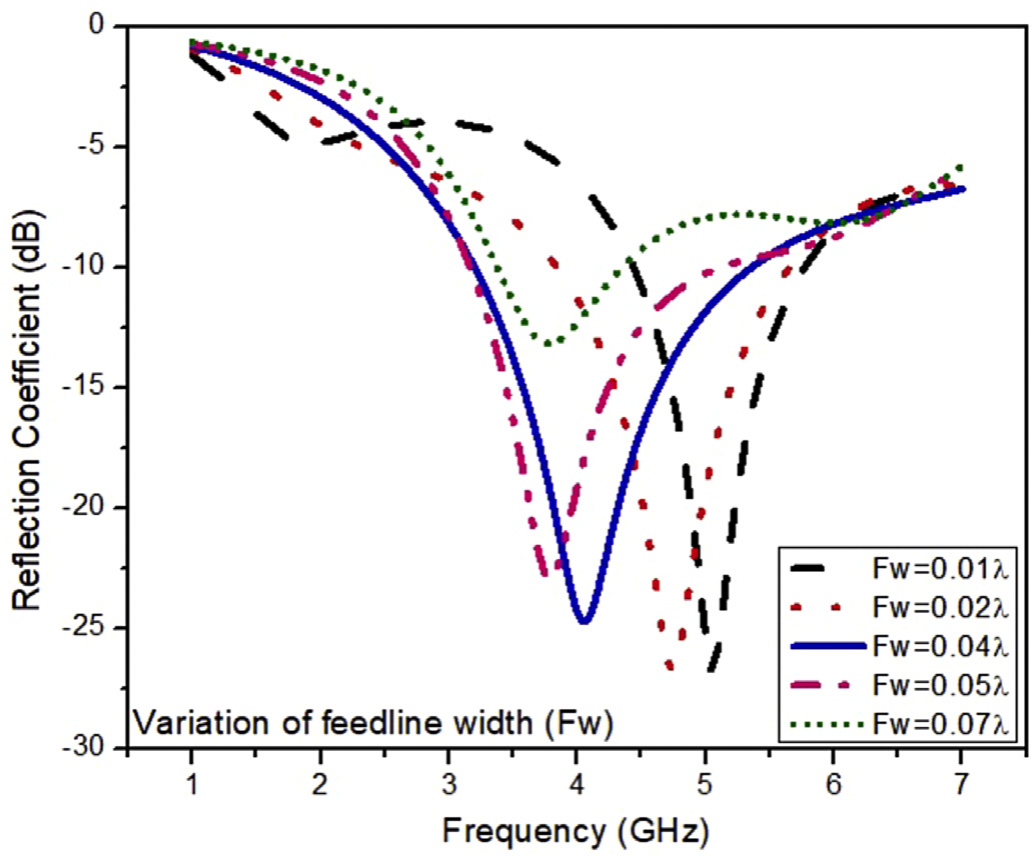
Effect of the width of the feed line (Fw) on the reflection coefficient of the ESSPR.

**Figure 10:**
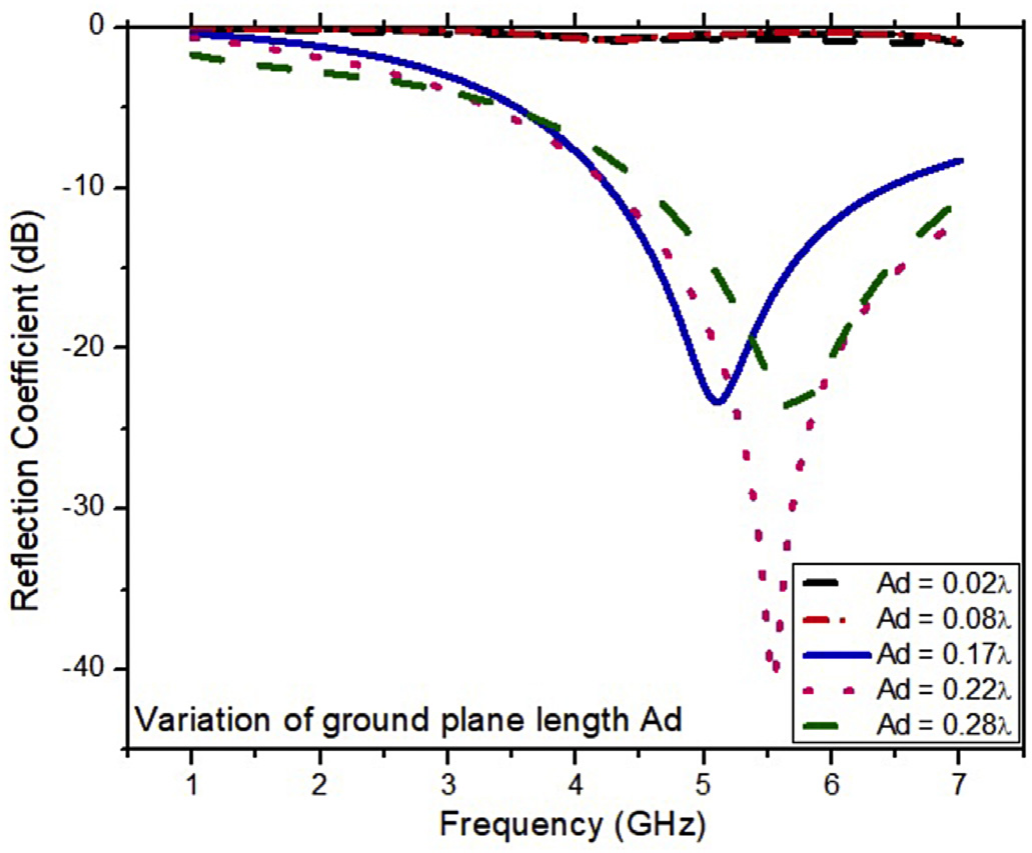
Effect of the length of the ground plane (Ad) on the reflection coefficient of the ESSPR.

The simulated results of the ESSPR are investigated and reported by examining the key geometrical parameters. The key geometrical parameters which are focused comprises of the radius of the semicircular patch (r), the major radius of the elliptical slot (Z), the length of the ground plane (Ad), and the width of the feed line (F_w_). The effect on the performance of the ESSPR by varying the radius of the semicircular patch (r) is shown in Figure 7. It shows that as the radius r is increased from the nominal value of 9.7 mm (0.13λ), the drift is seen on the higher end of the frequency spectrum with a decrease in the magnitude of the reflection coefficient up to -35 dB. Figure 8 shows the variation of the reflection coefficient with the radius of the major-axis (Z) of the elliptical slot. It can be observed from Figure 8 that the resonant frequency decreases on increasing the value of Z. Further, the effect of the width of the feed line on the reflection coefficient is recorded in Figure 9. As the width of the feed line (F_w_) is decreased, the reflection coefficient curve shifts to the higher end of the frequency spectrum with a slight increase in the amount of reflection. Also, as we increase the width of the feed line (F_w_), then the patch radiator lowers its capacity to transmit the radiation. The length of the ground plane is varied to report its effect on the reflection coefficient as shown in Figure 10. It is seen that upon decreasing the length of the ground plane from 12 mm, the antenna stops radiating. Also, as the length of the ground plane is increased above 12 mm, there is a sharp radiation response with drift in the frequency band towards the higher side. The optimum performance in terms of bandwidth within the sub-6 GHz frequency band is reported at the value of the length of the ground plane (Ad) of 12 mm (0.17λ).

The part of the ground plane is removed for improving the front to back ratio of the patch radiator by reducing the back lobe radiation. The parameters discussed are very important for fixing the band of radiation. The comparative analysis for all the design iterations is summarized in Table 3. It is seen that the ESSPR is of compact structure and provides the wide bandwidth with adequate radiation efficiency in the sub-6 ​GHz frequency range. The gain, directivity, and efficiency of ESSPR at different operating frequencies within the operating range are reported in Table 4.

**Table 3:** Various simulated parameters of various antenna configurations.

Parameter	Radiator3	Radiator4	Radiator5	Radiator6 (ESSPR)
W × L × h	0.34λ×0.34λ×0.02λ	0.34λ×0.34λ×0.02λ	0.34λ×0.34λ×0.02λ	0.34λ×0.34λ×0.02λ
Area of slot (mm^2^)	−	28.27	40	18.85
Bandwidth (MHz)	2410	1160	910	2140
FBW	53%	27.61%	22.66%	50.11%
Gain (dB)	1.81	2.98	2.78	2.76
Directivity (dB)	2.11	3.17	2.97	2.98
Radiation Efficiency (%)	94.5	96	95	96

**Table 4:** Simulated gain, directivity and radiation efficiency of the ESSPR.

Frequency (GHz)	Gain (dB)	Directivity (dB)	Radiation efficiency (%)
3.6	-0.07	0.11	95.98
3.8	0.37	0.56	96
4.0	0.79	0.98	95.78
4.2	1.18	1.38	95.58
4.4	1.54	1.74	95.48
4.6	1.88	2.09	95.38
4.8	2.20	2.41	95.29
5.0	2.49	2.70	95.21
5.2	2.76	2.98	95.13

## 4 Fabrication and measurement of the ESSPR

**Figure 11:**
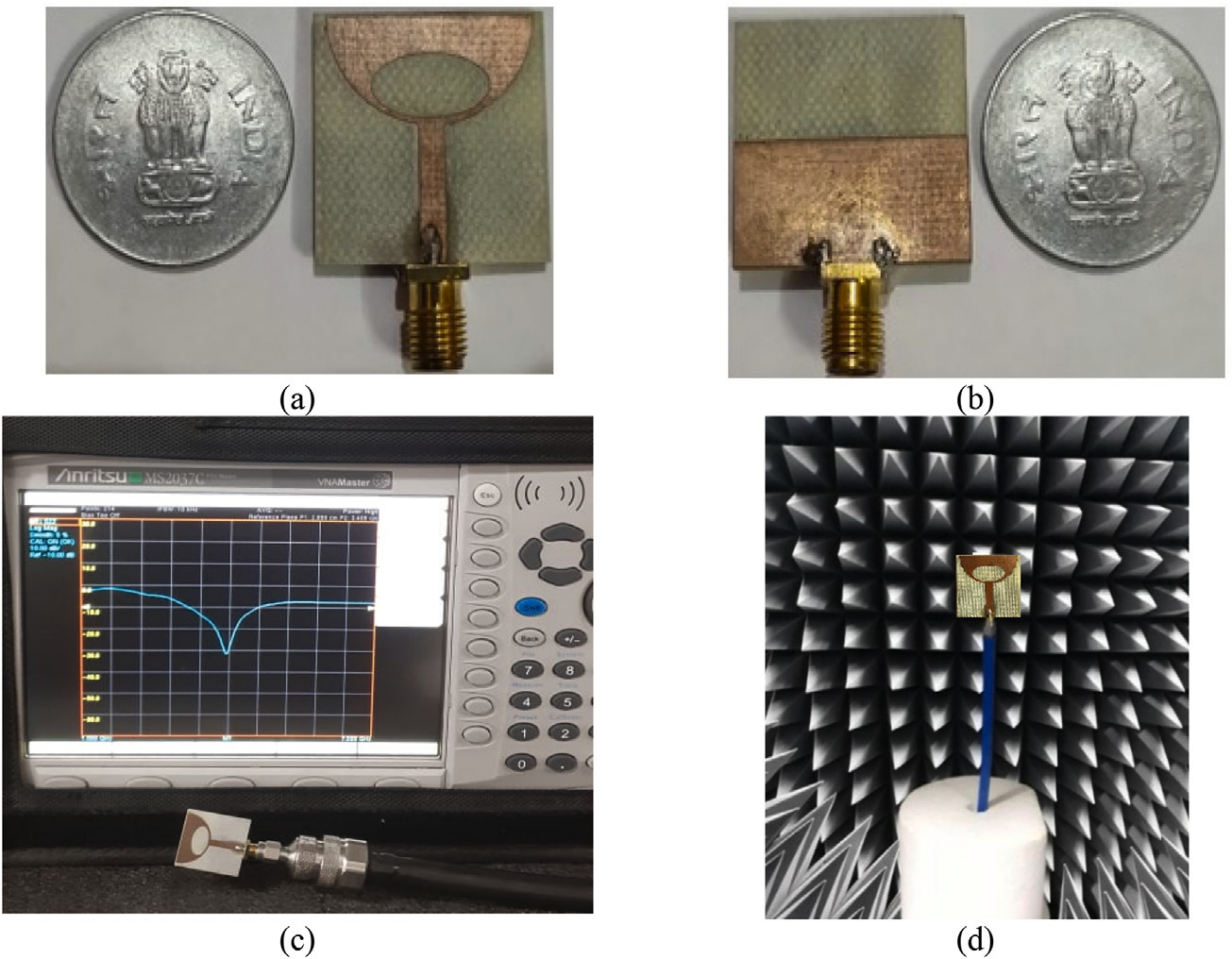
Fabrication and measurement of ESSPR, (a) front view of ESSPR prototype, (b) back view of ESSPR prototype, (c) reflection coefficient measurement setup, and (d) radiation characteristics measurement.

To validate the simulation results retrieved from the HFSS simulator for the designed ESSPR, the antenna is fabricated and measured. The top view and bottom view of the ESSPR prototype are shown in Figure 11(a) and Figure 11(b), respectively. The experimental setup for measuring the reflection coefficient using the vector network analyzer (VNA) is shown in Figure 11(c). The radiation characteristics measurement in anechoic chamber is shown in Figure 11(d).

**Figure 12:**
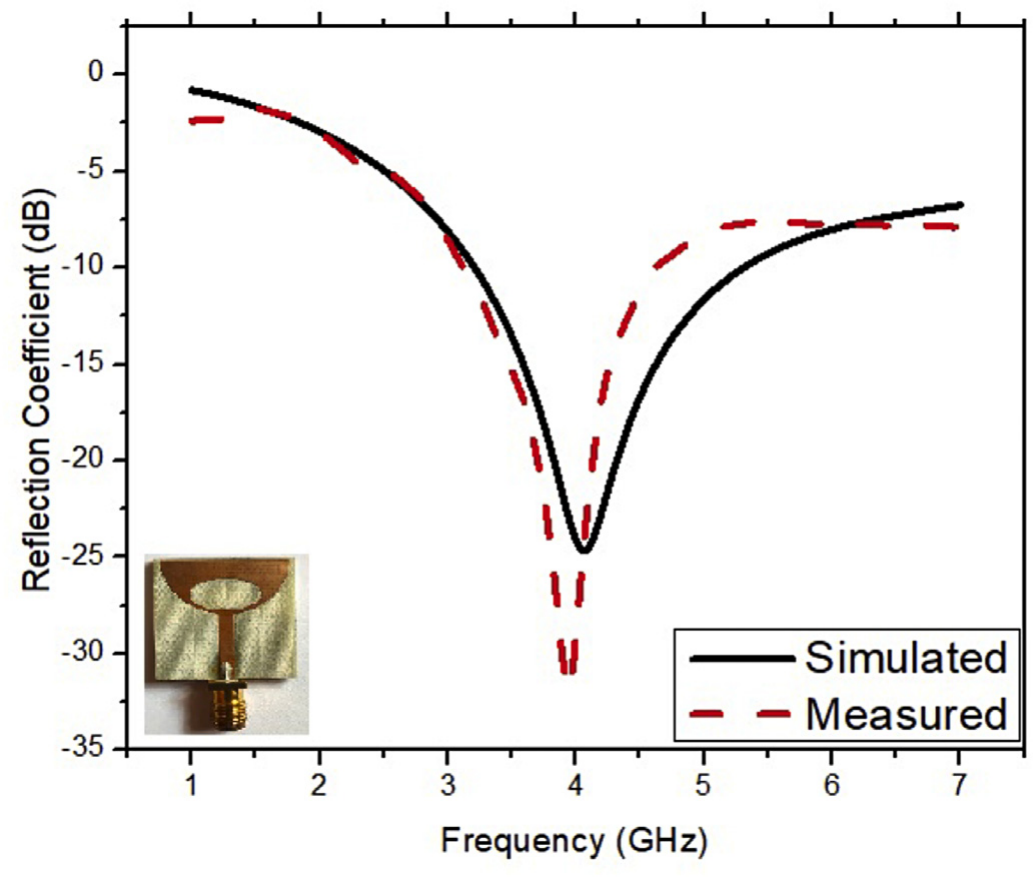
Comparison of measured and simulated reflection coefficient of the proposed ESSPR.

**Figure 13:**
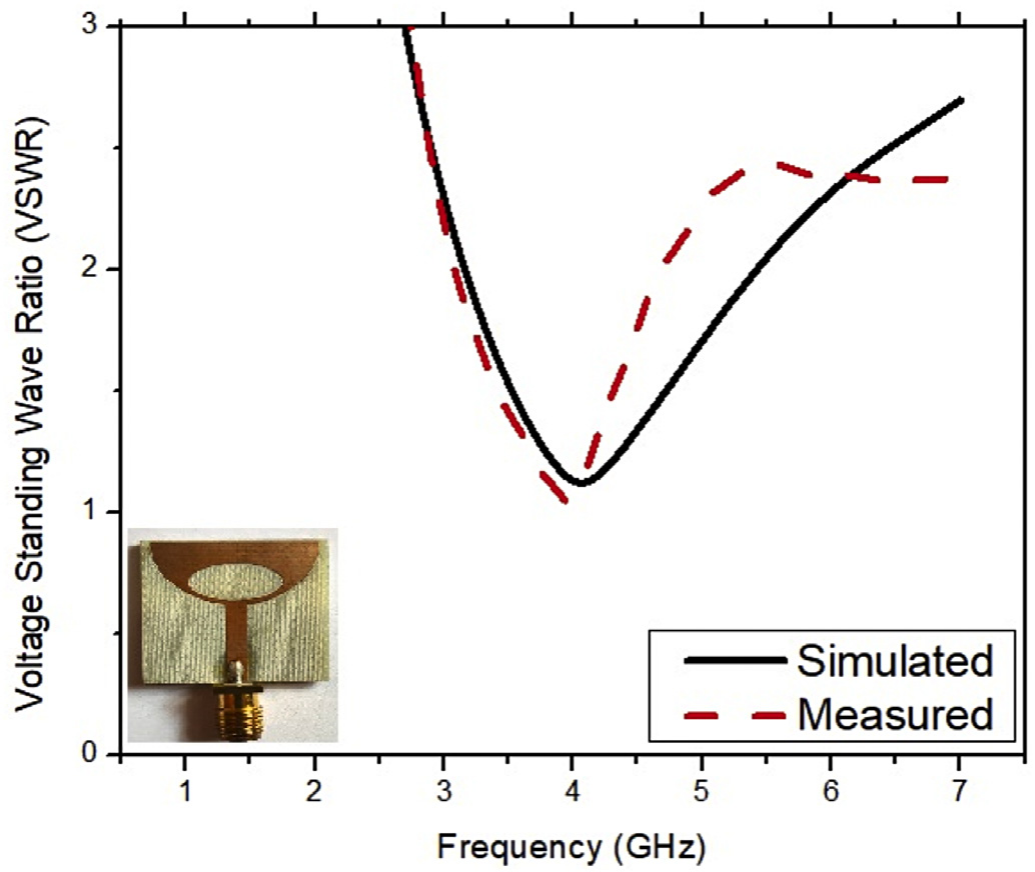
VSWR of the proposed ESSPR.

**Figure 14:**
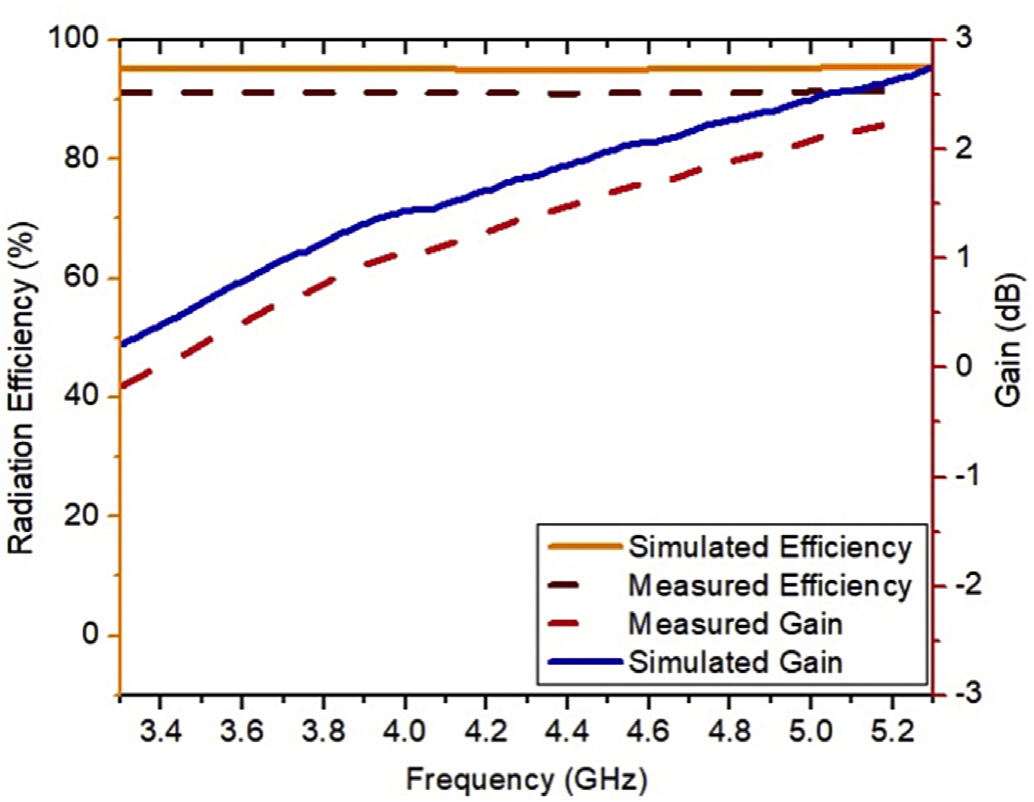
Gain and radiation efficiency of the proposed ESSPR.

The results retrieved from the experimental setup are compared with the simulation results as shown in Figure 12. It is observed from this figure that the results retrieved from the simulation and measurement setup show good agreement. The graph of the VSWR versus frequency of the ESSPR is illustrated in Figure 13. The operating frequency range, the fractional bandwidth and the minimum reflection coefficient obtained from the simulated results are 3.2–5.34 GHz, 50.11% and -24.64 dB, respectively. The operating frequency range, the fractional bandwidth and the minimum reflection coefficient obtained from the measured results are 3.14–4.72 GHz, 40.2% and -33.21 dB, respectively. The simulated and measured gain and radiation efficiency are shown in Figure 14. The minor variations might be due to fabrication errors and connector losses. The simulated maximum gain, simulated maximum radiation efficiency, measured maximum gain and measured maximum radiation efficiency of the ESSPR within the bandwidth are 2.76 dB, 96%, 2.45 dB and 92%, respectively. The results retrieved from the simulations and the experimental setup are compared in Table 5.

**Table 5:** Summary of the simulated and measured parameters of the ESSPR.

Parameter	Simulated	Measured
Highest frequency (f_H_) (GHz)	5.34	4.72
Lowest frequency (f_L_) (GHz)	3.20	3.14
Operating band (GHz)	3.20–5.34	3.14–4.72
Fractional bandwidth (%)	50.11	40.20
Maximum gain (dB)	2.76	2.45
Maximum radiation efficiency (%)	96	92
Minimum reflection coefficient (dB)	-24.64	-33.21

**Figure 15:**
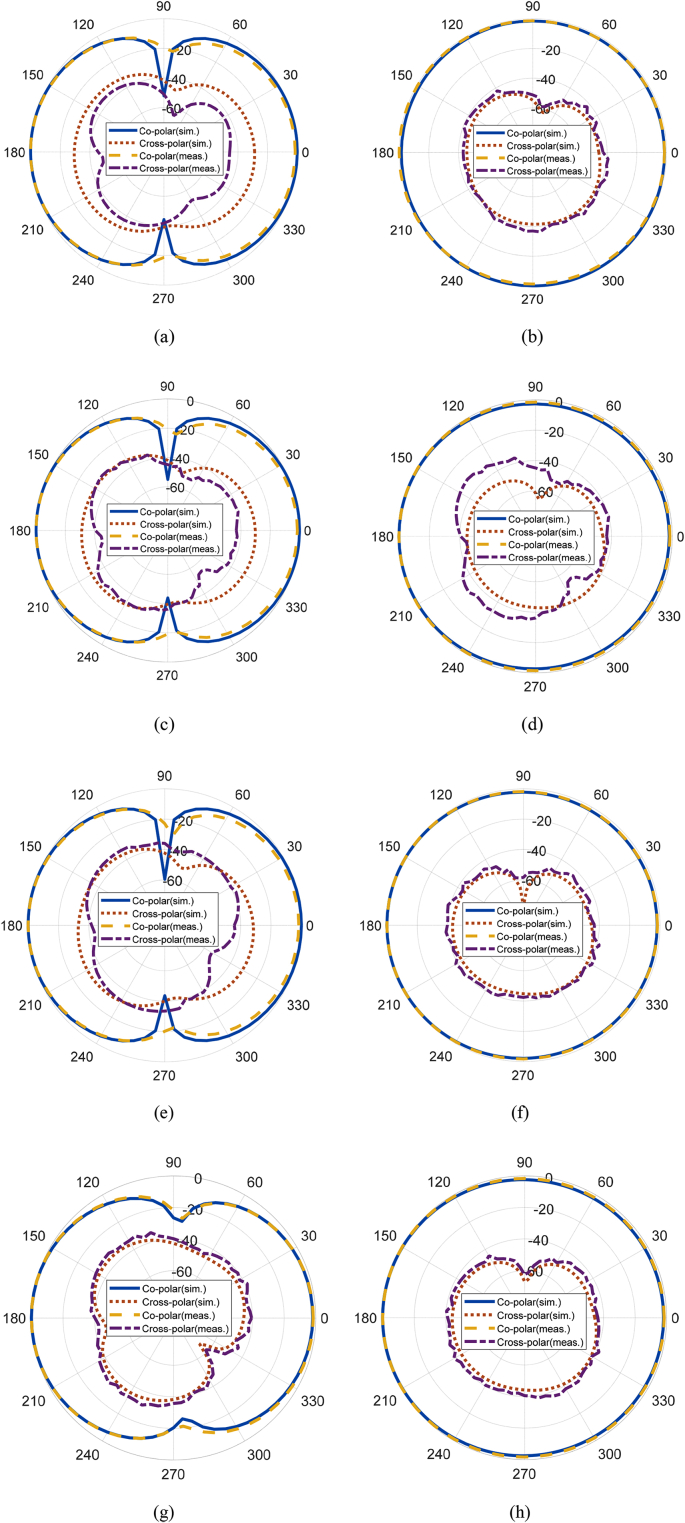
Simulated and measured normalized radiation patterns of the ESSPR,(a) E-plane (3.8 GHz), (b) H-plane (3.8 GHz), (c) E-plane (4 GHz) (d) H-plane (4 GHz), (e) E-plane (4.2 GHz), (f) H-plane (4.2 GHz), (g) E-plane (4.4 GHz) and (h) H-plane (4.4 GHz).

The normalized co-polar and cross-polar radiation patterns of the ESSPR in E-plane and H-plane are shown in Figure 15. From these patterns, it can be observed that the behavior of patterns are figure of eight and omnidirectional in E-plane and H-plane, respectively.

The proposed ESSPR offers miniaturization along with the wideband characteristics. The comparison of this design with the existing designs available in literature is shown in Table 6. The wavelength (λ) in the comparison table is calculated at the center frequency (${\text{f}}_{\text{c}}$) of 4.27 GHz. As observed from Table 6, the proposed ESSPR has a miniaturized area of (A_ESSPR_) 0.1156λ^2^, which is the smallest. The proposed compact ESSPR is suitable for sub-6 GHz wideband applications.

**Table 6:** Performance comparison of the proposed ESSPR with the existing designs in sub- 6 ​GHz range.

Ref.	Centre frequency (f_c_) (Bandwidth) (f_L_ – f_H_) (GHz)	Radiator type	ε_r_	Thickness	Gain (G) and Efficiency(η)	Physical size (L_sub_×W_sub_)	Radiator area	FBW(%)
[5]	3.65 (3.3–4.0)	Partial slotted ground RPA	4.4	0.01λ	G = 2.5dB, η = Not reported	0.48λ × 0.34λ	0.1632**λ**^**2**^	19.17
[6]	3 (2–4)	Monopole slot antenna	4.5	0.008λ	G = 4.55dB, η = 80%	0.5λ × 0.8λ	0.4 **λ**^**2**^	66
[7]	6.3 (3.281–7.45)	Pentagonal slot antenna	4.4	0.03λ	G = 4.24dB, η = 85%	0.52λ × 0.52λ	0.27 **λ**^**2**^	66
[13]	6.7 (4.69–8.71)	CPW fed T slot broadband monopole antenna	4.4	0.03λ	G = 3.31dB, η = 91.1%	0.94λ × 0.94λ	0.88**λ**^**2**^	60
[15]	4.9 (4.5−5.3)	Elliptical slot antenna	4.4	0.04λ	G = 10.3dB, η = Not reported	1.41λ×1.27λ	1.79 **λ**^**2**^	152
[17]	5.5 (4−7)	C-shaped patch antenna	4.4	0.02λ	G = 4.45dB, η = Not reported	0.55λ×0.73λ	0.40 **λ**^**2**^	54.54
[25]	10 (2–18)	Semi-circular floral shaped directional UWB antenna	4.4	0.05λ	G = 4.5dB, η = Not reported	1λ×1λ	λ^2^	160
[27]	5.76 (4–7.52)	Patch antenna with engraved elliptical slot	4.4	0.03λ	G = 3dB, η = 80%	0.95λ × 0.95λ	0.90 λ^2^	61
[28]	4.43 (3.05–5.82)	UWBcircular patch antenna	4.4	0.02λ	G = 0.34dB, η = 87.13%	0.29λ × 0.41λ	0.1189 λ^2^	62
[37]	2.366 (1.821–2.912)	Tuning fork stub based patch antenna	4.4	0.012λ	G = 4dB, η = Not reported	0.42λ × 0.42λ	0.1764 λ^2^	46.11
**Proposed ESSPR**	4.27 (3.20–5.34)	Elliptical slot based semi-circular patch radiator with partial ground	4.4	0.02λ	G = 2.76dB, η = 96%	0.34λ × 0.34λ	0.1156 λ^2^	50

## 5 Conclusion

A compact and miniaturized patch radiator for exhibiting wideband response in sub-6 GHz 5G frequency range has been designed and developed. An elliptical slot is engraved on the semicircular patch to achieve wide bandwidth i.e. from 3.2 GHz to 5.34 GHz. The designed antenna offers compact dimensions of 0.34λ × 0.34λ × 0.02λ, where λ represents the wavelength at the center frequency of the operating band. The proposed radiator finds its suitability for sub-6 GHz wideband applications. The measured results confirm the wide bandwidth of 2.14 GHz i.e. 50% fractional bandwidth. The simulated and measurement results show good agreement. The antenna parameters confirm that the designed and developed compact wideband ESSPR is suitable for 5G communication devices.

## Declarations

### Author contribution statement

Ankush Kapoor: Conceived and designed the experiments; Performed the experiments; Analyzed and interpreted the data; Wrote the paper.

Ranjan Mishra, Pradeep Kumar: Conceived and designed the experiments; Wrote the paper.

### Funding statement

This research did not receive any specific grant from funding agencies in the public, commercial, or not-for-profit sectors.

### Data availability statement

Data included in article/supplementary material/referenced in article.

### Declaration of interests statement

The authors declare no conflict of interest.

### Additional information

No additional information is available for this paper.
